# Frequency-Resolved High-Frequency Broadband Measurement of Acoustic Longitudinal Waves by Laser-Based Excitation and Detection

**DOI:** 10.3390/s24051630

**Published:** 2024-03-01

**Authors:** Felix Brand, Klaus Stefan Drese

**Affiliations:** Institute of Sensor and Actuator Technology, Coburg University of Applied Sciences and Arts, Am Hofbräuhaus 1b, 96450 Coburg, Germany; felix.brand@hs-coburg.de

**Keywords:** laser ultrasound, photoacoustics, frequency resolved phase velocity, attenuation, dispersion, laser ultrasonic spectroscopy, ndt, longitudinal waves, compression waves, acoustics

## Abstract

Optoacoustics is a metrology widely used for material characterisation. In this study, a measurement setup for the selective determination of the frequency-resolved phase velocities and attenuations of longitudinal waves over a wide frequency range (3–55 MHz) is presented. The ultrasonic waves in this setup were excited by a pulsed laser within an absorption layer in the thermoelastic regime and directed through a layer of water onto a sample. The acoustic waves were detected using a self-built adaptive interferometer with a photorefractive crystal. The instrument transmits compression waves only, is low-contact, non-destructive, and has a sample-independent excitation. The limitations of the approach were studied both by simulation and experiments to determine how the frequency range and precision can be improved. It was shown that measurements are possible for all investigated materials (silicon, silicone, aluminium, and water) and that the relative error for the phase velocity is less than 0.2%.

## 1. Introduction

In industry and academic research, optoacoustics has already been established in many applications [1]. The advantages of this method is the fact that the measurements are non-destructive and non-contact [1,2,3]. It should also be emphasised that photoacoustic measurements are very broadband due to laser excitation, which means that frequency-dependent analyses can be carried out in a range from kHz to GHz [4]. This allows macrostructures and thin films, as well as microstructures, to be probed [1]. Photoacoustics is, therefore, a promising technique for the study and analysis of complex media properties [3].

Bulk acoustic waves are typically non-dispersive, and measurements are performed with a single frequency or in a narrow frequency band. The recent literature has shown that the dispersion of bulk acoustic waves can be utilised to measure material properties such as material inhomogeneity. Karabutov et al. [5] presented a method that allows conclusions about the porosity of isotropic metal matrix composites from the dispersion of the phase velocity and the frequency-resolved attenuation of longitudinal waves. Podymova et al. [6] have demonstrated that the effect of porosity on the dispersion of longitudinal waves is also present in aluminium alloy matrix composites. Other researchers have characterised 3D-printed photopolymers by measuring frequency-resolved acoustic properties [7,8]. The dispersion of longitudinal waves inside of Polyvinylchloride (PVC) has been shown by Demcenko et al. [9].

In addition, during the development and testing of acoustic metamaterials, a frequency-resolved broadband characterisation of materials is necessary. Examples of metamaterials with dispersive longitudinal waves can be found in the literature [10,11,12]. Thus, broadband, frequency-resolved measurements of sound velocity and attenuation open up new possibilities for materials analysis.

One method of broadband excitation of acoustic waves is excitation via a nanosecond pulsed laser. This excitation method enables non-destructive and non-contact measurements with a broad frequency spectrum and high amplitude signals [13,14]. This excitation technique for short ultrasonic pulses is used for the excitation of longitudinal [15,16,17], shear [18,19,20], Rayleigh [21,22,23,24,25], and lamb waves [26,27,28,29]. All studies require high-quality experimental data and a deep understanding of the limitations of the experiment and the analysis.

In this paper, we present an optical method for precise, low-contact, non-destructive, sample-independent, broadband, and frequency-resolved measurement of the dispersion and attenuation of compression waves. The components of the measurement setup are introduced, and their purpose and impact on the measurement are discussed. Simulations and experiments were used to identify factors influencing the generation of sound by a pulsed laser. Further experiments were performed to analyse the accuracy of the method.

In the following section, we first introduce the idea of the measurement method and explain the components, starting with detection and going backwards to excitation. Special attention is paid to the measuring cell, its components, and its influence on the measurement. The influence on the measurement is first demonstrated using simulations and, later, using experimental measurements. The article concludes with dispersion measurements of various materials.

## 2. Materials and Methods

### 2.1. Concept of the Measurement Setup

**Figure 1:**
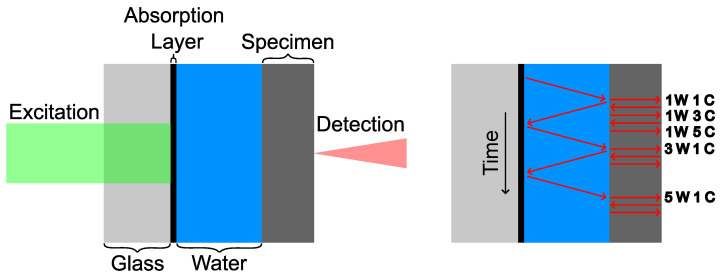
Scheme of acoustic measurement cell. (**Left**) Components of measurement cell. (**Right**) Scheme of possible acoustic paths. C: Path of compression wave through specimen. W: Path of compression wave through water. Time axis is not scaled.

The acoustic measurement cell is the centrepiece of the measurement setup. It consists of an absorption layer on top of a glass substrate, the specimen, and water in contact with the absorption layer and the specimen. There are several objectives that are addressed with the measuring cell presented below (see Figure 1):

- The measurement of the frequency-resolved phase velocity and attenuation of acoustic compression waves.
- The excitation and detection of an acoustic compression pulse independent of the sample material and over a wide frequency range.
- Non-destructive and ideally contactless measurement.
- The suppression of other acoustic excitations, e.g., shear waves.

**Figure 2:**
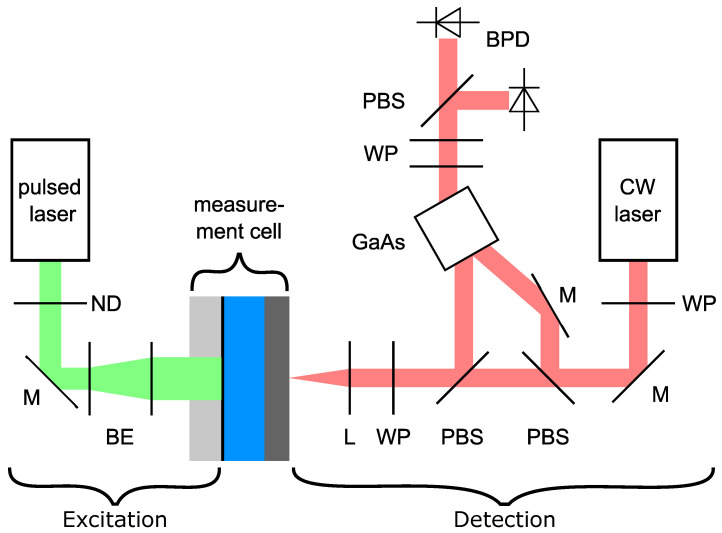
Complete measurement setup. Green: excitation, red: detection; ND: neutral density filter, BE: beam expander, M: mirror, WP: waveplate, PBS: polarizing beam splitter, L: lens, BPD: balanced photodetector.

The ultrasonic pulse is generated at the absorption layer via an ns pulsed laser. From this layer, the pulse travels through the water and the specimen and will be detected with an adaptive interferometer with a photorefractive crystal. The complete optical setup is visualised in Figure 2.

In the following section, we discuss the individual components, their purpose, and their influence on the measurement, starting at detection and going backwards to excitation.

### 2.2. Detection

The ultrasonic waves were detected using a self-built adaptive interferometer with a photorefractive crystal. These interferometers, used for the detection of ultrasonic waves or vibrations, are well described in the literature [30,31,32]. In our setup, we used an undoped GaAs crystal (5 × 5 × 10 ${\mathrm{mm}}^{3}$) in a diffusion-dominated regime with an anisotropic diffraction setup. The optical setup for the detection is shown in Figure 2.

This kind of interferometer has several advantages. The wavefront of the diffracted beam is identical to the wavefront of the nondiffracted beam of the sample beam, allowing both beams to interfere with exactly the same wavefronts [30,33]. A possible source of wavefront distortion can be, for example, the surface roughness of the sample. The dynamic hologram inside the crystal acts as a high-pass filter with a cut-off frequency $f_{c}$ with a time constant τ of the crystal ($f_{c}=1/\left(2\pi \tau \right)$). The cut-off frequency depends on the setup and is in the range of 3.5 and 10 kHz for GaAs crystals [30]. The high-pass suppresses low-frequency disturbances, such as air fluctuations, thermal deformation, acoustic noise, and other sources of vibration [30,33]. In addition the interferometer has a wide frequency response [33], with a flat transfer function above its cut-off frequency and below the bandwidth of the balanced photodetector [32,34].

Detection is the main component, which defines the noise level of the setup. The noise level is mainly determined by the laser power of the interferometer, the number of averages, the detector, and the electronic and acoustic background noise.

In our setup, we used a 1064 nm laser with a maximum CW power of 10 W (Azurlight Fiber Laser, Azurlight Systems, Bordeaux, France), operating at 3 W, and an InGaAs photodetector (Femto HBPR-200M-30K-IN, FEMTO Messtechnik GmbH, Berlin, Germany), with a bandwidth of DC up to 200 MHz.

### 2.3. Measurement Cell

This section describes the individual components of the measuring cell (see Figure 1) and their influence on the measurement.

#### 2.3.1. Specimen

The measuring cell was designed in such a way that the measurement is as sample-independent as possible.

It is only necessary for the evaluation (see Section 2.4.1) that two reflections of the same pulse (e.g., 1W 1C (the pulse travels one time through the water and the compression wave travels one time through the sample) and 1W 3C, as compared in Figure 1) can be measured. This sets the requirements for the sample in terms of acoustic attenuation and thickness.

#### 2.3.2. Fluid

The sound pulse is transmitted through the liquid to the sample. Only longitudinal waves can propagate in the liquid, which means that primarily longitudinal waves can be observed in the sample.

A further task of the fluid is to delay the reflection of the wave at the absorption layer (3W 1C, as compared in Figure 1) so that at least a number of $n_{S}$ reflections can be measured within the sample before the first interfering reflection occurs. This delay can be adjusted by the fluid thickness $x_{F}$ and its velocity $c_{F}$.

(1) $$x_{F}>c_{F}\;\cdot \;\left(n_{S}\;\cdot \;\frac{x_{S}}{c_{S}}+\frac{\tau_{\mathrm{Pulse}}}{2}\right),$$

where $x_{S}$ and $c_{S}$ are the thickness and acoustic velocity of the sample, and $\tau_{\mathrm{Pulse}}$ is the acoustic pulse duration. The first term of the equation describes that $n_{S}$ reflections can be measured before the wave propagating in the fluid is detected for the first time. The second term ensures that the two waves do not overlap due to the pulse duration.

Considering the acoustic attenuation in the fluid, the aim should be to minimise the fluid thickness while still fulfilling the condition above. The literature typically describes the frequency-dependent attenuation α by a power law [35,36]. For water, a quadratic dependence on frequency is commonly assumed [35,37,38,39,40,41,42].

(2) $$\alpha_{\mathrm{Water}}=k\;\cdot \;f^{2}$$

The literature often provides values for *k* in the range of 0.2 $\frac{\mathrm{dB}}{m{\mathrm{MHz}}^{2}}$, measured for frequencies up to 15 MHz [37,39,40,41]. Other sources give slightly higher attenuations of 0.4 $\frac{\mathrm{dB}}{m{\mathrm{MHz}}^{2}}$ to 1 $\frac{\mathrm{dB}}{m{\mathrm{MHz}}^{2}}$ [43] or <1 $\frac{\mathrm{dB}}{m{\mathrm{MHz}}^{2}}$ [44] up to very high values of 44 $\frac{\mathrm{dB}}{m{\mathrm{MHz}}^{2}}$ [36]. Sometimes, a linear dependence is also assumed with values from 0.2 $\frac{\mathrm{dB}}{m\mathrm{MHz}}$ [45] to 30 $\frac{\mathrm{dB}}{m\mathrm{MHz}}$ [46].

The reflection and transmission factor and, therefore, the acoustic impedance difference at the fluid–sample interface influence the signal strength of the different reflections inside the sample. On the one hand, a low reflection coefficient is good for the transmission of the acoustic wave from the fluid into the sample, and on the other hand, a high reflection coefficient is good for additional reflections within the sample. Neglecting the transmission between the sample and the surrounding air, the optimal reflection coefficient $R_{\mathrm{opt}}$ for the $n_{S}$ reflection inside the sample is

(3) $$R_{\mathrm{opt}}=\frac{n_{S}-1}{n_{S}}$$

This fact must be considered when selecting the liquid. The choice of liquid can be influenced by other factors, such as potentially hazardous substances, solvent vapours, and the material compatibility of the sample with the liquid. Equally important is the cleaning effort required to remove the liquid from the cell and the sample.

We used deionised water and hydraulic oil in our setup. Unless stated otherwise, the fluid layer was water and 9 mm thick.

#### 2.3.3. Absorption Layer

The absorption layer is the main part that influences the generation of acoustic waves. Excitation via an absorption layer has two advantages. Firstly, the material of the absorption layer can be freely chosen. Secondly, the excitation of the acoustic wave is independent of the sample.

The absorption layer should not be damaged; therefore, the excitation had to be selected to be in the thermoelastic regime (see Section 2.3.5). The damage threshold of the absorption layer defines the maximum pulse energy of the laser (Equation (7)).

The acoustic impedance of the absorption layer $Z_{AL}$ should match the impedance of the fluid $Z_{F}$ to enable good transmission of the acoustic pulse into the fluid.

(4) $$Z_{AL}\approx Z_{F}$$

In Section 3.1, we simulated the influence of different material properties on the elongation and frequency spectra of the excited acoustic pulse (see Formulas (19)–(21)). The elongation of the pulse increases with the thermal expansion coefficient while decreasing with the density, specific heat capacity, Young’s modulus, and optical absorption coefficient. The material parameters of polymer-based materials, with the exception of specific heat capacity and absorption coefficient, result in higher acoustic amplitudes compared to metal-based materials. Polymer-based materials also fulfil the impedance condition mentioned above better than metallic materials.

To achieve measurements with a high bandwidth, the decrease in the frequency spectrum should be small. According to the simulations (see Formula (20)), a high acoustic velocity and high optical absorption are advantageous. The desired slow decrease in the frequency spectrum is, therefore, inverse to the elongation in terms of the density and optical absorption coefficient. The frequency spectra (see Appendix A) show that the increasing power compensates for the stronger drop at high frequencies. Polymer-like materials are, therefore, preferable.

Another aspect to consider is the thickness of the absorber. If the absorber is slightly transparent, an acoustic pulse will be generated at the glass–absorption layer interface and the absorption layer–fluid interface. The influence of reflections within the absorption layer can be minimised by a layer that is as thin as possible.

We used two kinds of absorption layers. The aim of the first coating was to obtain a thin layer and a small slope in the frequency spectrum. A sputtering process was chosen for this purpose. Based on the absorption coefficient at the excitation wavelength, the coefficient of thermal expansion, the specific heat capacity, and the density, chromium was chosen for the absorption layer. The thickness was 125 nm.

The aim of the second layer was to excite waves with high amplitudes. Polydimethylsiloxane (PDMS) was chosen because of its very high coefficient of thermal expansion, low density, and low elastic modulus compared to metals. Carbon was added to the PDMS to increase optical absorption. The spin-coated layer was approximately 50 μm thick.

#### 2.3.4. Glass

A glass substrate has several functions. It acts as a support for the absorption layer and also as a constraining layer (see Section 2.3.5). A constraining layer increases the amplitude of the generated acoustic pulse. The glass needs to be transparent for the wavelength of the pulsed laser. The glass substrate must have a minimum thickness so that the reflection at the rear end of the substrate is sufficiently delayed until the wave reaches the detector position.

As discussed earlier, we want a minimum of $n_{S}$ pulses inside the sample before the first unwanted signal occurs. This results in a minimum glass thickness $x_{G}$ of

(5) $$x_{G}>\left(n_{S}-1\right)x_{S}\;\cdot \;\frac{c_{G}}{c_{S}}+c_{S}\;\cdot \;\tau_{\mathrm{Pulse}}$$

where $c_{G}$ and $c_{S}$ are the longitudinal velocity inside the glass and sample, respectively; $x_{S}$ is the thickness of the sample; and $\tau_{\mathrm{Pulse}}$ is the acoustic pulse duration. The signal strength is also positively influenced by a high reflection factor between the glass and the absorption layer. A high impedance difference should be aimed for.

(6) ZG≫ZAL or ZG≪ZAL

where $Z_{G}$ and $Z_{AL}$ are the acoustic impedance of the glass and the absorption layer.

#### 2.3.5. Excitation

For laser excitation, there are several points to consider: the excitation regime, the excitation spot size, the laser wavelength, and the pulse duration.

There are two regimes for laser excitation of ultrasonic waves. The first is thermoelastic excitation, which is truly non-destructive because the absorbed laser pulse is below the damage threshold of the material [15]. The second regime is ablative excitation, which is destructive because the laser pulse energy is above the damage threshold and vaporises the material [15].

Aussel et al. [47] show that material evaporation due to excessively absorbed power densities AW occurs under the following condition:

(7) $$AW\geq \frac{\pi }{4}\sqrt{\frac{\lambda_{th}\rho c_{p}}{\tau_{L}}\left(T_{v}-T_{i}\right)}$$

where $\lambda_{th}$ is the thermal conductivity, ρ is the density, $c_{p}$ is the specific heat capacity, $\tau_{L}$ is the laser pulse duration, $T_{v}$ is the vaporisation temperature, and $T_{i}$ is the initial temperature.

For a point laser source, the excited waves under a propagation angle θ can be described by analytical formulas [13].

For the thermoelastic excitation of a point source [13]:

(8) $$\begin{array}{c} u_{c,\mathrm{thermoelastic}}∼\frac{\sin2\theta \;\cdot \;{\left(k^{2}-\sin^{2}\theta \right)}^{0.5}}{{\left(k^{2}-2\sin^{2}\theta \right)}^{2}+4\sin^{2}\theta {\left(1-\sin^{2}\theta \right)}^{0.5}\;\cdot \;{\left(k^{2}-\sin^{2}\theta \right)}^{0.5}}\\ u_{s,\mathrm{thermoelastic}}∼\frac{k\sin4\theta }{k{\left(1-2\sin^{2}\theta \right)}^{2}+4\sin^{2}\theta {\left(1-\sin^{2}\theta \right)}^{0.5}\;\cdot \;{\left(1-k^{2}\sin^{2}\theta \right)}^{0.5}} \end{array}$$

**Figure 3:**
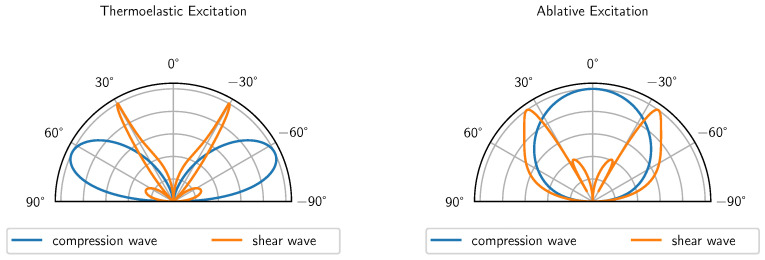
Directivity of compression and shear waves for point sources in case of aluminium.

And, for the ablative excitation of a point source [13]:

(9) $$\begin{array}{c} u_{c,\mathrm{ablative}}∼\frac{2k^{2}\cos\theta \left(k^{2}-2\sin^{2}\theta \right)}{{\left(k^{2}-2\sin^{2}\theta \right)}^{2}+4\sin^{2}\theta {\left(1-\sin^{2}\theta \right)}^{0.5}\;\cdot \;{\left(k^{2}-\sin^{2}\theta \right)}^{0.5}}\\ u_{s,\mathrm{ablative}}∼\frac{\sin2\theta {\left(1-k^{2}\sin^{2}\theta \right)}^{0.5}}{k{\left(1-2\sin^{2}\theta \right)}^{2}+4\sin^{2}\theta {\left(1-\sin^{2}\theta \right)}^{0.5}\;\cdot \;{\left(1-k^{2}\sin^{2}\theta \right)}^{0.5}} \end{array}$$

where the wavevector $k=c_{c}/c_{s}$. The index *c* represents the compression wave, and the index *s* represents the shear wave. The analytical formulas are visualised in Figure 3 for aluminium.

The measuring cell presented in this paper is based on compression waves that propagate in a normal direction. Therefore, a point source in the thermoelastic excitation regime is not suitable due to the directivity of the compression waves (see Figure 3 (left)). Ablative excitation has a suitable radiation characteristic but cannot be used because of the minor surface damage it causes, which would damage the thin absorption layer.

**Figure 4:**
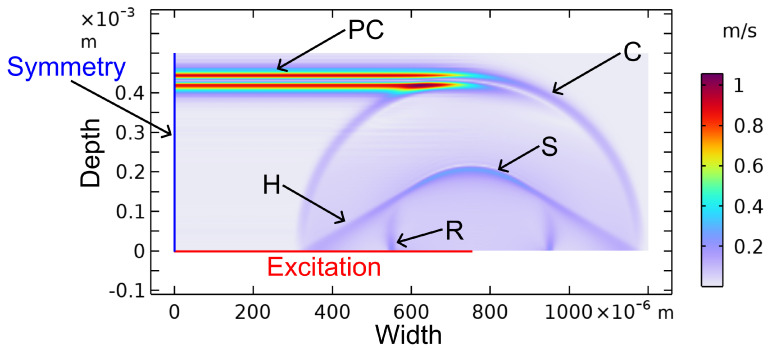
Simulation of excited waves in aluminium via heat impulse after 75 ns. C: compression wave; S: shear wave; R: Rayleigh wave; H: head wave; PC: plane compression wave. Colour scale corresponds to particle velocity.

Since the use of point sources is not advantageous in our case, extended sources were investigated. The excited waves using extended sources can be calculated using FEM. In our case, we used COMSOL 6.1. The propagated waves after 75 ns are visualised in Figure 4. The extended source results in a plane compression wave (PC) as well as additional waves (head (H), compression (C), shear (S), and Rayleigh (R) waves) at the edge of the excitation [47]. These waves do not contribute to the sensing concept but can interfere with the plane compression waves.

A plane compression wave, whose plane wave front propagates parallel to the excitation, is the desired wave for this application. This plane compression wave is not obtained in point excitation.

In addition, the strength of the excitation amplitude can be increased by using a transparent constraining layer, resulting in a buried ultrasonic source. This enables the generation of normal stresses, resulting in an enhanced generation of compression waves [48]. Hutchins [49] used light oil, silicone resin, water, and acetone as a constraining layer and increased the compression wave amplitude by 21 to 25 dB compared to the unmodified surface. The use of a glass slide, cemented to the surface, increased the amplitude by 30 dB. The use of a buried source is, therefore, advantageous and was used in the experiments. In our case, the glass substrate and the water acted as a constraining layer. Deionised water was used to minimise the interaction with the specimen and reduce the cleaning effort compared to other fluids like oil.

If the measurement is not completely in the near field of the acoustic waves, there are two negative aspects to consider. In the far field, sound energy decreases with distance *z* by $\propto 1/z^{2}$ [50], while it remains constant in the near field. In addition, the ultrasonic wave experiences a phase shift of π/2 in the transition between near and far field [51,52].

If the waves were measured in the transition area or in the far field, both effects had to be taken into account when calculating the attenuation or phase velocity. The near-field length *L* can be used as an approximation for the transition between both fields [53,54].

(10) $$L=\frac{D^{2}}{4\;\cdot \;\lambda }$$

where *D* represents the excitation diameter, and λ represents the wavelength of the acoustic wave. High-frequency components are normally in the near field and therefore not affected. The transition to the far field for low-frequency components resulted in a lower cut-off frequency, at which errors occurred.

The laser wavelength was of secondary importance. The laser light should have low absorption in the glass and high absorption in the absorption layer.

**Figure A6:**
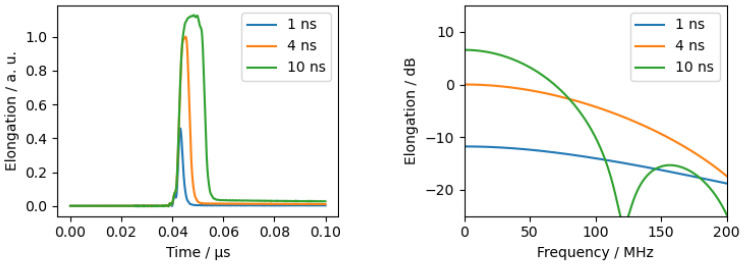
FEM-Simulation with different heat pulse length.

Pulse duration influenced the amplitude and frequency spectra of the excited waves. Longer pulses resulted in a higher energy input into the absorption layer and increased the amplitude of the acoustic wave while decreasing the frequency bandwidth (Formulas (20) and (21) and Figure A6). For measurements in the mid to upper double-digit MHz range, a single digit pulse duration of a few nanoseconds was appropriate. Short pulses with high power may damage the sample.

In our experiments, we used a 4 ns pulsed laser (Brilliant, Quantel-Laser, Les Ulis Cedex, France) at 532 nm and a pulse energy of about 18 mJ (after some neutral density filter). The beam diameter was chosen to be 18 mm to ensure diffraction-free propagation of the acoustic beam. A second pulsed laser (Wedge, Bright Solutions, Cura Carpignano, Italy) with a 1 ns pulse duration, at 532 nm, and a 1 mJ pulse energy was used to investigate the influence of pulse duration. Due to the low pulse energy, a beam cross-section of 2 mm was selected for this laser.

### 2.4. Data Analysis Methods

As mentioned in the introduction, the target quantity is a frequency-resolved measurement of phase velocity and attenuation over a wide frequency range. This chapter presents a method for calculating phase velocity and attenuation, determining an SNR level, and a method for correcting diffraction.

#### 2.4.1. Determination of Phase Velocity

One method for calculating a frequency-resolved phase velocity $c_{ph}$ is a phase spectrum analysis (PSA). This method uses two signals of the same acoustic wave $s_{1}$ and $s_{2}$ with a certain path difference Δx and compares the phase change between both signals. This is achieved with the following formula [55,56]:

(11) $$c_{ph}\left(f\right)=\frac{2\pi f\Delta x}{\Delta \phi }.$$

The phase difference Δϕ can be calculated from the Fourier-transformed signals of both windowed signals [56]:

(12) $$\Delta \phi =\arctan\frac{I\left(F\left(s_{1}\right)\;\cdot \;F{\left(s_{2}\right)}^{*}\right)}{R\left(F\left(s_{1}\right)\;\cdot \;F{\left(s_{2}\right)}^{*}\right)}$$

where F is the operator for the fast Fourier transformation, ∗ is the operator for the complex conjugated value, and I and R are operators for the imaginary and real parts, respectively.

#### 2.4.2. Determination of Noise Level

**Figure 5:**
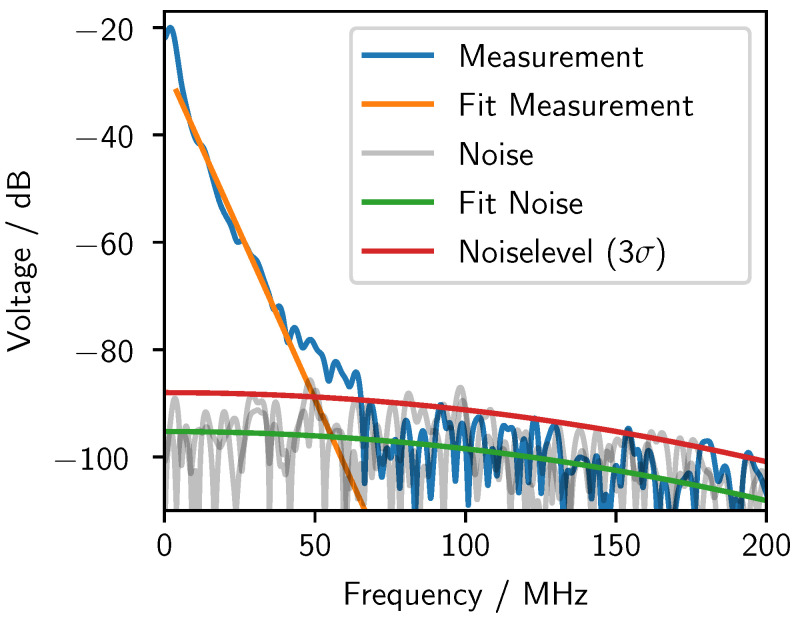
Determination of noise level from numerous noise measurements (grey signals) and the quadratic fit. The intersection of the noise level and the linear fit of the measurement data defined the maximum frequency of the signal.

To decide up to which frequency there was still sufficient signal strength, a frequency-dependent noise level was calculated by measuring noise $S_{\mathrm{noise}}$ without any acoustic signal. The noise spectrum is Gaussian-like and is determined by the measurement setup and the digital filter used. The noise spectrum N(f) is calculated by

(13) $$N\left(f\right)=20\mathrm{dB}\log_{10}\left|F(S_{\mathrm{noise}})\right|$$

several times, and a quadratic formula was fitted to the averaged noise level. Three times the standard deviation of the fit residuals on top of the quadratic fit indicates the border between noise and signal (Figure 5).

The signal $S_{\mathrm{measurement}}$ can be approximated by a linear decay in the logarithmic frequency spectrum [57]. Low frequencies are neglected in the linear fit due to diffraction effects (see Section 2.4.4). The intersection between the linear approximation of the signal and the approximation of the noise level $N_{\mathrm{noiselevel}}$ defined the bandwidth of the signal. Every frequency above was interpreted as noise and was neglected.

The energy spectra shown in Section 3 are plotted as SNR.

(14) $$SNR=S_{\mathrm{measurement}}-N_{\mathrm{noiselevel}}$$

#### 2.4.3. Determination of Attenuation

In addition to the phase velocity, it was also possible to calculate the frequency-resolved attenuation α of the material within the path Δx.

(15) $$\alpha =\frac{20\mathrm{dB}\log\frac{\left|F(s_{1})\right|}{\left|F(s_{2})\right|}}{\Delta x}$$

For the attenuation, the bandwidth was defined by the SNR of both signals $s_{1}$ and $s_{2}$.

Within the setup described in Section 2.1, the analysed ultrasound waves were reflected at the sample–air interface and the sample–water interface. These reflections and the associated energy transfer to the water and air had to be taken into account when calculating the attenuation. The energy remaining in the sample depends on the reflection coefficient *R* [58]

(16) $$R={\left(\frac{Z_{2}-Z_{1}}{Z_{1}+Z_{2}}\right)}^{2}$$

where the acoustic impedance $Z=\rho \;\cdot \;c_{ph}$. Each reflection results in an offset of the attenuation spectra and needs to be corrected. This results in the following formula

(17) $$\alpha =\frac{20\mathrm{dB}\log\frac{\left|F(s_{1})\right|}{\left|F(s_{2})\right|}+\sum_{i}10\mathrm{dB}\log\left(R_{i}\right)}{\Delta x}$$

where *i* represents the reflections that occur between the signals $s_{1}$ and $s_{2}$ of the acoustic pulse.

#### 2.4.4. Diffraction Correction

For small beam diameters, long propagation paths, or low frequencies, the acoustic wave can transition from the near to the far field. This transition occurs at the approximated near field length *L* (see Equation (10)).

A phase difference of π/2 of the acoustic wave between the near and far field distorts the determination of the phase velocity. The calculation of the attenuation in the far field is also subject to errors, as the energy decreases with distance. A correction of the phase and amplitude can be performed by calculating the diffracted wave A(x,y,z) [52]

(18) $$A\left(x,y,z\right)=\frac{i}{\lambda z}\exp\left(-\frac{i\pi }{\lambda z}\left(x^{2}+y^{2}\right)\right)\;\cdot \;F\left(A_{0}\left(x,y\right)\exp\left(-\frac{i\pi }{\lambda z}\left(x^{2}+y^{2}\right)\right)\right)$$

where *z* is the propagation direction, $A_{0}\left(x,y\right)$ is the acoustic source distribution, and *i* is the imaginary unit. To the best of our knowledge, there is no better correction for the approach taken.

## 3. Results and Discussion

With the use of simulation, we investigated the influence of the material properties of the absorption layer and the laser parameters on the excitation of the acoustic waves. These results were verified by experiments. The phase velocities of various materials were determined from further measurements.

### 3.1. Simulation of Material and Laser Influence on the Excitation

Simulations were carried out with the aim of characterising the influence of the material parameters of the excited object to maximise the strength of the planar compression wave. The second criterion was a high-frequency bandwidth of the acoustic pulse to maximise the frequency range from which later information can be deduced. The simulations were 2D FEM simulations (similar to Figure 4), employing both the structural mechanics and heat transfer modules from COMSOL software. (V 6.1) Both were coupled using the formula for thermal expansion.

The optical excitation was simulated by a time-dependent heat flux along the excitation line of 750 μm and an exponential decay of the heat flux in the depth direction with a damping factor $\alpha_{op}$. The thermal pulse had a constant intensity, a duration $\tau_{L}$ of 4 ns, and was completely absorbed. Different from what is usually found in the literature, we calculated the acoustic pulse in a solid absorption layer instead of a weakly absorbing fluid [59,60].

The simulations used aluminium as the material, with the parameters given in Table 1. The simulation area was larger than the penetration depth of the optical pulse.

In the simulations, the material and optical parameters were varied to determine the influence on the excited acoustic wave in the time and frequency domain. The optical parameters were laser pulse length $\tau_{L}$ and optical material attenuation $\alpha_{op}$. The laser power was kept constant as the pulse length was varied. The material parameters (thermal conductivity $\lambda_{th}$, coefficient of thermal expansion $\alpha_{th}$, specific heat capacity $c_{p}$, density ρ, and Young’s modulus *E*) were varied to cover the entire range of common materials. Appendix A contains the simulated signals and shows the dependence of these parameters on the sum of the squared elongations $s_{ac}^{2}$, which is proportional to the energy of the excited planar compression wave.

(19) $$s_{ac}^{2}\propto \frac{\alpha_{th}^{2}\;\cdot \;\tau_{L}^{1.5}}{c_{p}^{2}\;\cdot \;\alpha_{op}^{1.5}\;\cdot \;\rho^{1.25}\;\cdot \;E^{0.75}}$$

For short optical pulses, the linearly scaled frequency spectrum of the elongations can be approximated using a Gaussian function [57]. The standard deviation σ and the amplitude *A* of the Gaussian function $A\exp\left(-f^{2}/\sigma^{2}\right)$ of the autocorrelated elongations were determined. This led to the following expressions for the plane compression wave:

(20) $$\sigma \propto \sqrt{\frac{\alpha_{op}}{\tau_{L}}\;\cdot \;\sqrt{\frac{E}{\rho }}}$$

(21) $$A\propto \frac{\alpha_{th}^{2}\;\cdot \;\tau_{L}^{2}}{c_{p}^{2}\;\cdot \;\alpha_{op}^{2}\;\cdot \;\rho \;\cdot \;E}$$

The formula for the squared elongations in the time domain is identical to the integral of the Gaussian function. The aim was to obtain a high elongation and a high σ for high-quality broadband measurements. A higher bandwidth can be achieved by a higher acoustic velocity ($c\propto \sqrt{\frac{E}{\rho }}$), stronger optical absorption, or shorter pulse durations. With the exception of the thermal expansion coefficients and the specific heat capacity, the energy cannot be optimised independently of the bandwidth. In fact, an optimisation of $s_{ac}^{2}$ can simultaneously cause a worsening of σ. However, a smaller σ can often be neglected due to the higher elongations (see spectra in Appendix A).

### 3.2. Characterisation of Acoustic Measurement Cell

Measurements were carried out to characterise the effect of the laser pulse width, the fluid (width and type) and the excitation layer.

#### 3.2.1. Excitation Layer

**Figure 6:**
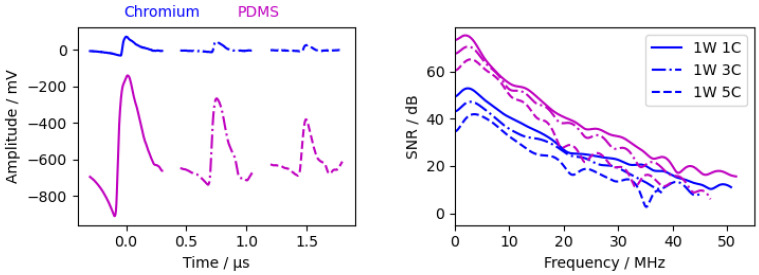
Measurements with different excitation layers. (**Left**) time signal from interferometer. PDMS signals have an offset of −610 mV. (**Right**) calculated SNR according Section 2.4.2.

As mentioned in Section 2.3.3, measurements were carried out with two different excitation layers ( 125 nm chromium, 50 μm PDMS). The results are shown in Figure 6.

The material parameters of the PDMS layer were not known, so a comparison with Formulas (19)–(21) cannot be made. Nevertheless, it is clear that the waves excited by the PDMS layer have a higher amplitude due to the different material parameters, probably mainly due to the higher coefficient of thermal expansion and the lower specific heat capacity, Young’s modulus, and density. The amplitude of the PDMS layer is about 7 to 9 times higher than the chromium layer. As long as the SNR level is high enough, the calculated phase velocity is independent of the excitation layer.

**Figure 7:**
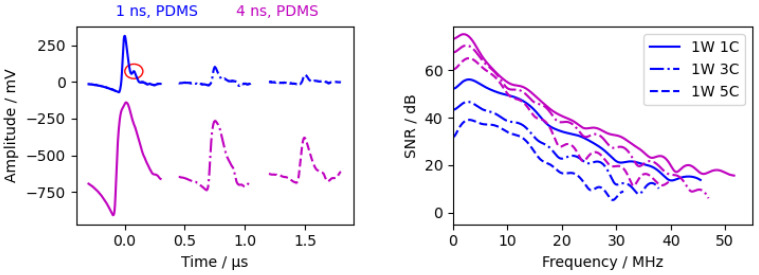
Measurements with different excitation layers. (**Left**) time signal from interferometer. The 1 ns signal was amplified by 3, the 4 ns signals have an offset of −610 mV, and the red circle shows the reflection within the PDMS layer. (**Right**) calculated SNR according Section 2.4.2.

The decrease in signal strength with higher frequencies is higher for the PDMS layer than for the chromium layer. This is predicted by the simulations and Formula (20). The lower amplitude combined with the slower decrease in signal strength resulted in a similar maximum frequency for PDMS and chromium. The waviness in the PDMS spectrum is caused by an additional wave within the signal peak. The additional wave can be seen clearer at the second peak in the time domain. This wave is even more visible in the measurement with a 1 ns pulsed laser (Figure 7, red circle). The reason for this additional wave is the thicker PMDS layer in which the wave is reflected.

Normally, a superposition of two waves is not desired for the phase spectrum analysis. It is, therefore, preferable to use the chromium layer as long as the amplitudes are sufficiently high.

#### 3.2.2. Laser Pulse Width

In addition to the 4 ns pulse laser, we performed measurements with a 1 ns pulse laser ( 1 mJ pulse energy). Due to the lower pulse energy, the beam diameter had to be reduced to 2 mm to observe an acoustic wave (the 4 ns laser had a beam diameter of 18 mm). The measurements are shown in Figure 7. For better visibility, the measurement signal from the 1 ns laser is amplified by a factor of 3.

Due to the shorter 1 ns pulse, the additional reflection within the PDMS layer was clearly visible in the 1W1C signal in the time domain (Figure 7, red circle). The time delay between the two peaks is 0.076 μs, corresponding to a PDMS layer thickness of 42 μm. The subsequent waves decay rapidly and are barely observed. The decay between the different waves is greatly enhanced due to the small beam diameter and, therefore, the high diffraction of the acoustic pulse. The lower amplitude and slower decay in the frequency domain are analogous to the simulations (see Figure A6). The frequency spectrum of the 1W1C wave had a similar bandwidth between the different pulse lasers. This suggests that the acoustic bandwidth can be further increased by using a higher pulse energy and a 1 ns pulsed laser.

This suggests that the bandwidth could be further increased with a 1 ns laser pulse if the laser has a higher pulse energy.

#### 3.2.3. Water Layer Thickness

**Figure 8:**
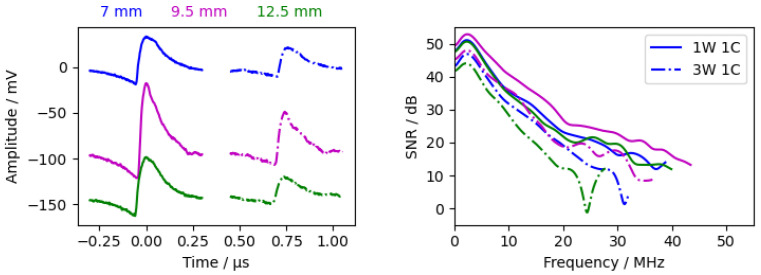
Measurements with different water layer thicknesses with chromium as excitation layer. (**Left**) time signal from the interferometer. The 9.5 mm measurement has an offset of −90 mV, and the 12.5 mm measurement has an offset of −140 mV. (**Right**) calculated SNR according Section 2.4.2.

**Figure 9:**
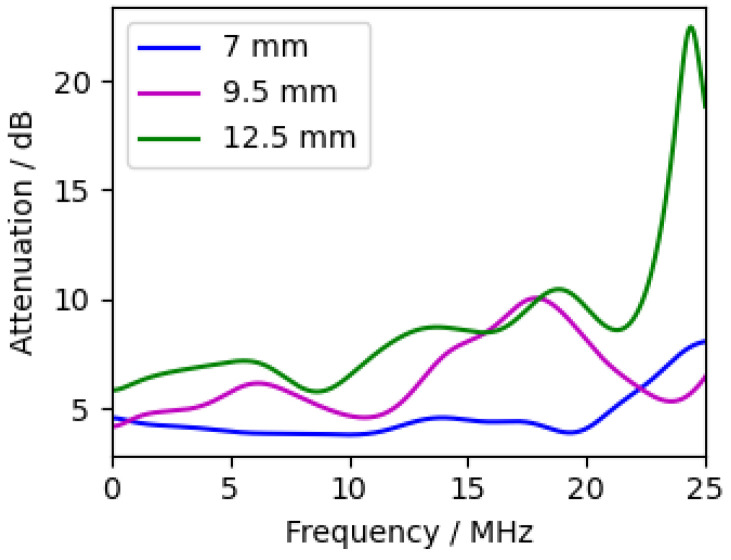
Frequency-resolved attenuation of measurements with different water layer thicknesses (Equation (15)). Compared peaks are 1W1C and 3W1C. Excitation layer was chromium.

Figure 8 shows three measurements with different water layer thicknesses (with chromium as the excitation layer). The measurements showed an unexpected behaviour, as the amplitudes are significantly higher with a 9.5 mm water layer than with a 7 mm water layer, although the attenuation in the 9.5 mm measurement should be higher. In the calculated attenuation spectrum (Figure 9), the attenuation between 1W1C and 3W1C increased with increasing water thickness. Therefore, the unexpected behaviour is probably due to different levels of excitation due to laser power fluctuations. In summary, the water layer should be as thin as possible but thick enough to clearly separate the waves within the sample and the waves within the water.

#### 3.2.4. Type of Fluid

**Figure 10:**
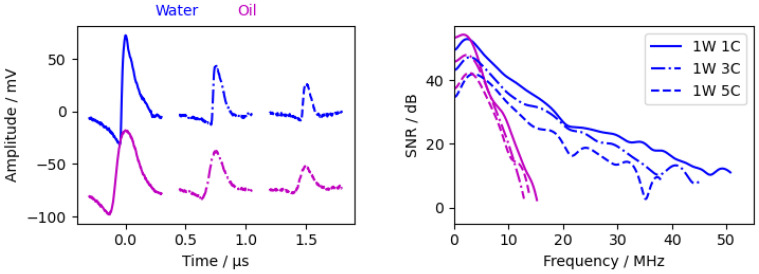
Measurements with oil and water between excitation layer and specimen. Chromium was used as excitation layer. (**Left**) time signal from the interferometer. Oil signals have an offset of −70 mV. (**Right**) calculated SNR according to Section 2.4.2.

The last variable investigated was the fluid between the excitation layer and the sample. The water was replaced by a mineral hydraulic oil (HLP 46, viscosity 46 $\frac{{\mathrm{mm}}^{2}}{s}$), and the results are shown in Figure 10. The oil significantly attenuated higher frequencies, whereas lower frequencies (<3.5 MHz) were amplified by up to 3 dB. This suggests that oil may be a better choice for low-frequency measurements but is not suitable for frequencies above 5 MHz. This is consistent with the literature, in which it has been reported that water typically has an attenuation of ∼0.2 $\frac{\mathrm{dB}}{{\mathrm{MHz}}^{2}m}$ [37,39,40] and mineral oil has an attenuation of 24 $\frac{\mathrm{dB}}{{\mathrm{MHz}}^{2}m}$ [61].

### 3.3. Frequency Resolved Measurements in Different Materials

In this section, we show that frequency-resolved phase velocity measurements were possible in virtually any material. For this reason, we have chosen a high-velocity material (silicon single crystal), a metal (aluminium), a soft low-velocity material (silicone), and a liquid (water).

#### 3.3.1. Silicon

**Figure 11:**
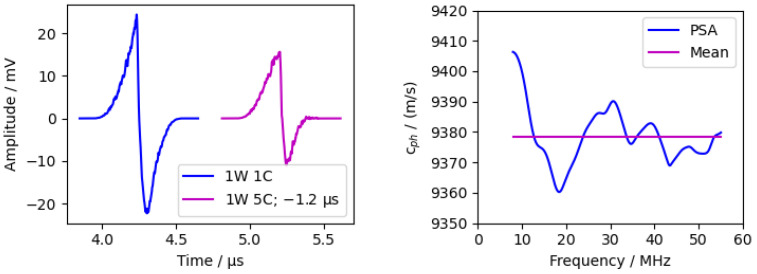
Measurement of 5 mm thick silicon sample. (**Left**) time domain with shifted second peak. (**Right**) phase velocity calculated via phase spectrum analysis (PSA).

The sample used was a silicon window with a 25.4 mm diameter and a 5 mm thickness. The acoustic pulse travelled along the 5 mm axis, which corresponds with the [111]-orientation of the silicon crystal. The measurement is visualised in Figure 11. The PDMS absorption layer was used.

The calculated phase velocity was approximated constant over the frequency with a mean value of 9378.4 $\frac{m}{s}$ and a standard deviation of 9.2 $\frac{m}{s}$.

The anisotropic phase velocity of silicon crystal in the [111]-direction in material libraries vary from 9362 $\frac{m}{s}$ [62] to 9372 $\frac{m}{s}$ [63].

Considering the inaccuracy of the thickness measurement (using a micrometre screw), the measurements were in good agreement with the literature values. The percentage deviations were 0.17% [62] and 0.07% [63].

#### 3.3.2. Aluminium

**Figure 12:**
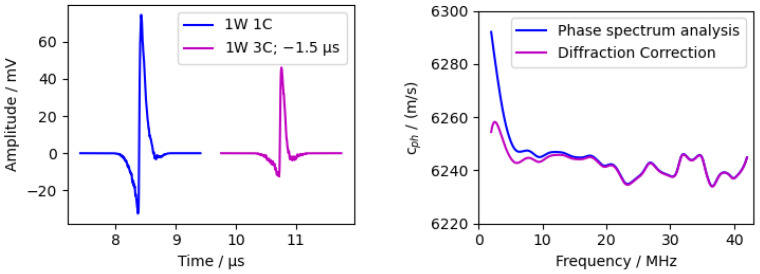
Measurement of 12 mm thick aluminium sample. (**Left**) time domain with shifted second peak. (**Right**) phase velocity calculated via phase spectrum analysis (PSA).

The aluminium sample was 12 mm thick in the measurement direction and wide enough to suppress any reflections from the sample boundaries. The absorption layer used was chromium. The results are shown in Figure 12.

The phase velocity showed a constant value for frequencies above 9 MHz with an increasing velocity towards lower frequencies. This was due to the transition between the near and far field and the resulting phase change of π/2. The diffraction correction (Section 2.4.4) significantly reduces the increase at low frequencies and, thus, improves the result. No comparison was made between the measured sound speed and the literature data, as the literature data differ greatly due to the different alloys used (from 6200 $\frac{m}{s}$ to 6400 $\frac{m}{s}$ [35]).

#### 3.3.3. Silicone

The silicone sample was 2.2 mm thick and translucent. The silicone used was SF00 from “Silikonfabrik”. The absorption layer used was PDMS.

**Figure 13:**
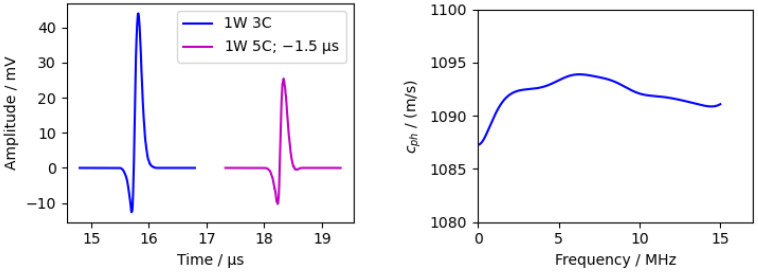
Measurement of 2.2 mm thick silicone sample. (**Left**) time domain with shifted second peak. (**Right**) phase velocity calculated via phase spectrum analysis (PSA).

As can be seen in Figure 13, measurements in the soft silicone layer were possible up to a frequency of 15 MHz. The measurement was made more challenging by the fact that the acoustic impedance of water and silicone is very similar; therefore, only a small proportion of the sound pulse was reflected at the interface (R~2%). This resulted in a low SNR and a low maximum frequency. With a velocity of ~1090$\frac{m}{s}$, the measurements agree quite well with other measurements of similar materials in the literature [64,65,66].

#### 3.3.4. Water

**Figure 14:**
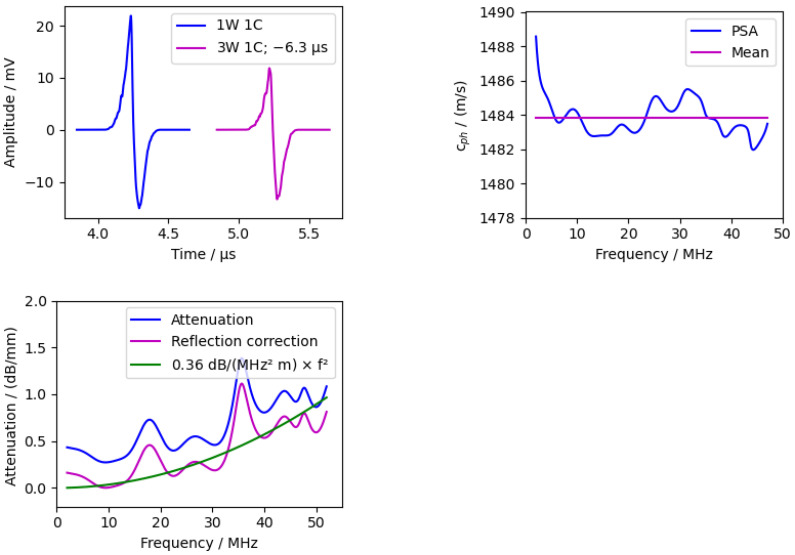
Measurement of 5.4 mm thick water layer between PDMS absorption layer and silicon window. (**Left**) time domain with shifted second peak. (**Right**) phase velocity calculated via phase spectrum analysis (PSA). (**Bottom**) attenuation of water with quadratic fit.

In addition, the fluid between the excitation layer and the sample can be analysed. The measurement of the silicon sample was used for this, and the reflections in the water were analysed. The results are shown in the following figures (Figure 14).

The mean velocity was 1483.8 $\frac{m}{s}$ with a standard deviation of 1 $\frac{m}{s}$. The literature value for water at 20 °C is 1482.3 $\frac{m}{s}$ [67]. Considering the accuracy of the thickness measurement and the temperature uncertainty, the measurement is in good agreement.

In contrast to the previous measurements, the attenuation is also shown, as there are literature values for water with which our measuring cell can be compared. The “Reflection correction” takes into account the reflection coefficients (see Section 2.4.3). As mentioned in Section 2.3.2, the attenuation in water is usually described by a quadratic dependence on the frequency. The attenuation coefficients vary in a wide range between 0.2 $\frac{\mathrm{dB}}{m{\mathrm{MHz}}^{2}}$ [37,39,40,41] and 1 $\frac{\mathrm{dB}}{m{\mathrm{MHz}}^{2}}$ [43,44] and up to 44 $\frac{\mathrm{dB}}{m{\mathrm{MHz}}^{2}}$ [36].

The measurement presented here had an attenuation coefficient of 0.36 $\frac{\mathrm{dB}}{m{\mathrm{MHz}}^{2}}$, which is between the lower and slightly higher literature values.

## 4. Conclusions

In this work, we presented a method to measure the longitudinal phase velocity and attenuation contactless over a high-frequency range (~3 to 55 MHz, depending on material). We have investigated all components of the acoustic measuring cell and identified their influencing factors on the measurement. Simulations were used to analyse the excitation of compression waves. An unfocused beam with a large beam diameter is required to excite the intended planar compression waves. For the excitation of pulses with high energies, an absorption layer with a low elastic modulus, low density, low specific heat capacity, and high thermal expansion should be chosen. However, these material parameters also cause the energy of the excited acoustic waves to decrease more sharply at higher frequencies (Section 3.1). We were able to show this behaviour in a simulation and an experiment.

Therefore, polymers with additives to increase optical absorption would be suitable to excite high-energy waves. The greater decrease in energy at higher frequencies for polymers is partially offset by the increased energy. However, the experiments have shown that polymers need to be applied as a thin layer while still having a high optical absorption to suppress additional reflections of the acoustic pulse at the polymer–water and polymer–glass interfaces. These additional pulses interfere with the determination of the phase velocity and attenuation. The thin sputtered chromium layer ( 125 nm) did not show the additional pulse, but the amplitude decreased by a factor of 7 to 9 in comparison with the PDMS layer. Future experiments should, therefore, consider a new polymer (-like) material for the absorption layer, which can be applied thinly and has a high optical absorption.

The water layer fulfilled the intended purpose: transmitting only the compression wave, enabling the excitation of broadband acoustic pulses independent of the sample material, and separating the waves in the time domain. The interferometer with a photorefractive crystal also showed that it can measure the acoustic pulse independent of the sample material up to high frequencies, even with weakly reflective samples.

The accuracy of the setup was demonstrated by measuring silicon and water, the sound velocities of which have been extensively analysed in the literature. The relative error was below 0.2%. The accuracy of the attenuation measurement cannot be estimated since there are no good reference materials with consistent attenuation values found in the literature. Nevertheless, the attenuation measurement for water is between the attenuation values specified in the literature.

In summary, we have developed a measurement technique that can measure the frequency-resolved phase velocity and attenuation of a sample contactlessly (only in contact with water), non-destructively, independently of the sample, and over a wide frequency range.

## Figures and Tables

**Table 1:** Used material properties for the FEM simulation.

Elastic modulus *E*	70 GPa
Density ρ	2700 $\frac{\mathrm{kg}}{m^{3}}$
Poisson ratio ν	0.33
Coefficient of thermal expansion $\alpha_{th}$	$23\times 10^{-6}$ $\frac{1}{K}$
Thermal conductivity $\lambda_{th}$	238 $\frac{W}{\mathrm{mK}}$
Specific heat capacity $c_{p}$	900 $\frac{J}{\mathrm{kg}K}$

**Figure A1:**
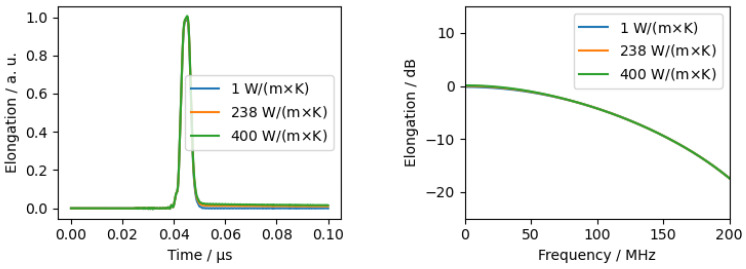
FEM-Simulation with different thermal conductivity.

**Figure A2:**
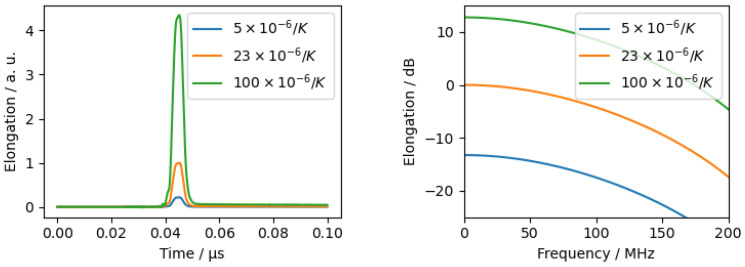
FEM-Simulation with different coefficient of thermal expansion.

**Figure A3:**
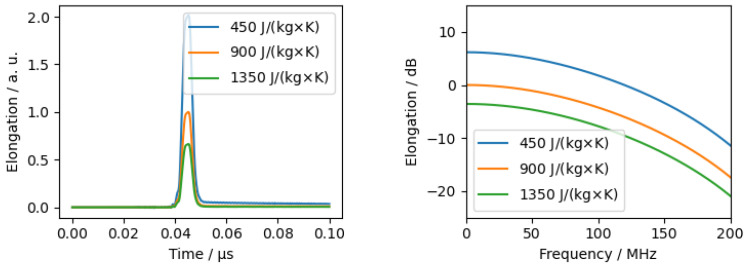
FEM-Simulation with a different specific heat capacity.

**Figure A4:**
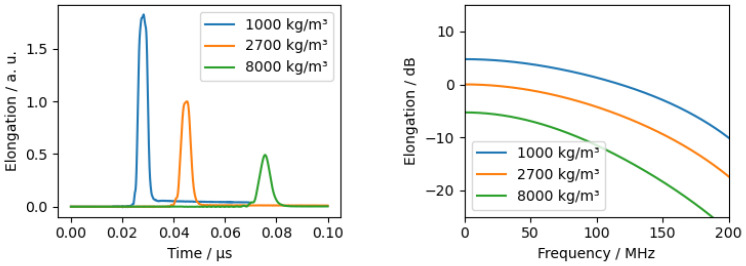
FEM-Simulation with different densities.

**Figure A5:**
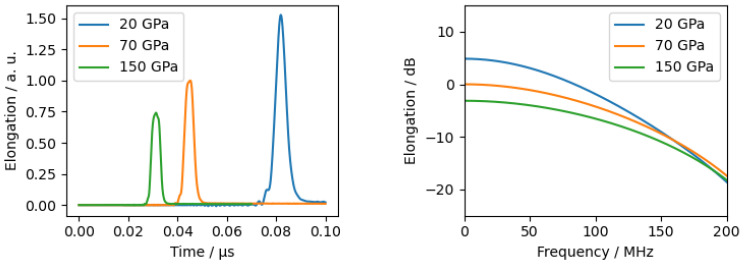
FEM-Simulation with different Young’s moduli.

**Figure A7:**
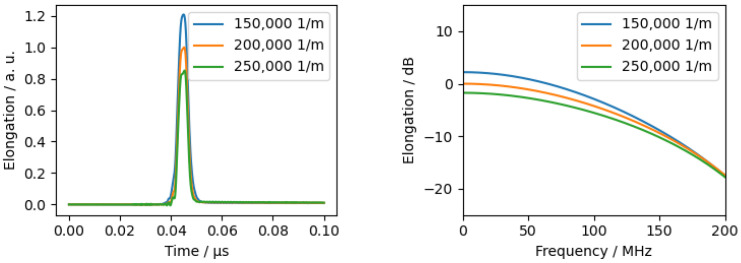
FEM-Simulation with different material absorption coefficient.

**Figure A8:**
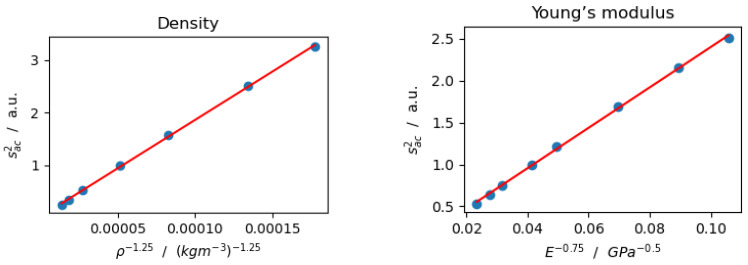

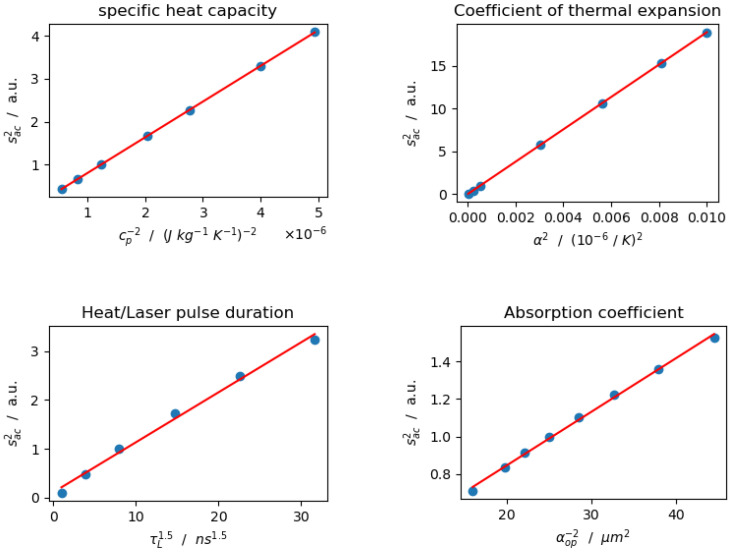
Dependencies of material and excitation parameters on the elongation $s_{ac}^{2}$.

**Figure A9:**
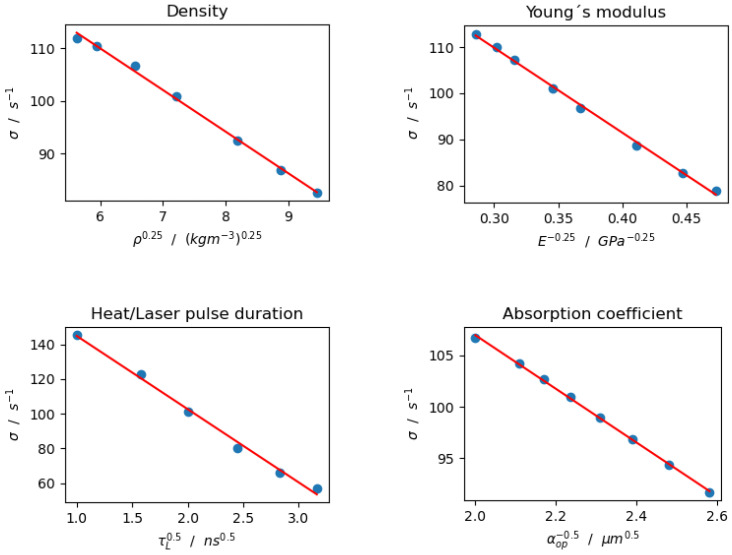
Dependencies of the material and excitation parameters on the σ parameter.

**Figure A10:**
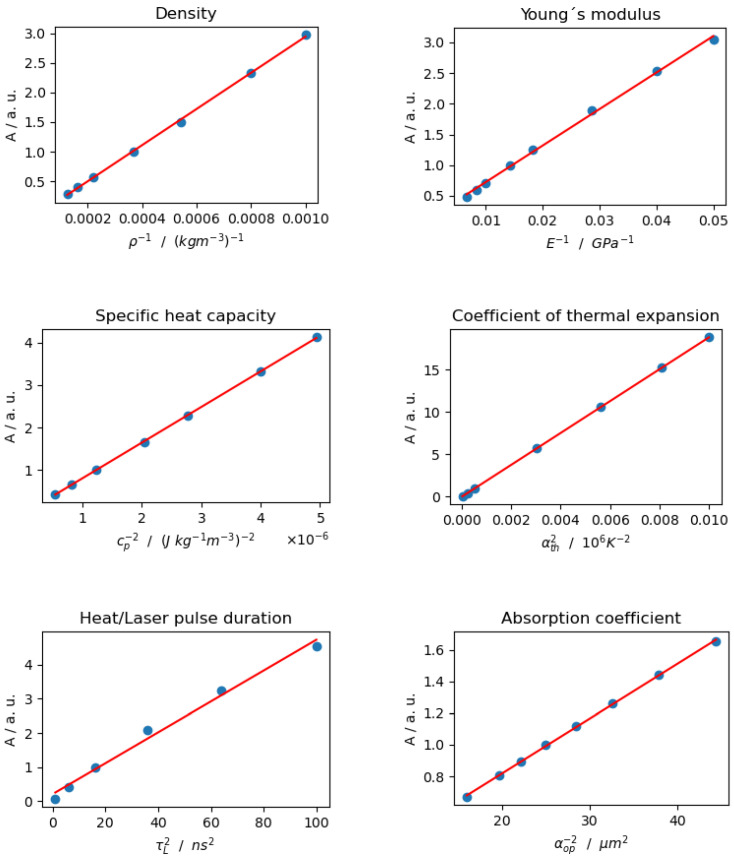
Dependencies of material and excitation parameters on the *A* parameter.

## Data Availability

The data presented in this study are available on request from the corresponding author.

## Appendix A. Simulation of Laser-Based Excitation of Ultrasonic Waves

This appendix details the simulations in Section 3.1 to characterise the influence of the absorption layer.

Simulations were performed in which different material and laser properties were varied in order to observe the effect on the amplitude and the frequency spectrum of the excited acoustic compression pulse. The material parameters were selected to cover the entire range of common materials.

The time signals of the elongation on the symmetry axis in a depth of 0.25 mm is plotted. The elongations of the time signals are scaled to the elongation of the original material properties (see Table 1). The frequency spectra of the pulses are equally normalised to the original material parameters.

The influence of the thermal material properties (thermal conductivity, coefficient of thermal expansion, and specific heat capacity) can be seen in Figure A1, Figure A2 and Figure A3. The thermal conductivity has no influence on the elongation nor the frequency spectrum. This is due to the fact, that the heat diffusion is rather slow compared to the penetration depth, the duration of the time of the heat pulse and the acoustic wave propagation. This results in an almost stable temperature profile.

The thermal expansion and the specific heat capacity had a strong effect on the excited elongation $s_{ac}^{2}\propto \alpha_{th}^{2}/c_{p}^{2}$. Both were quite intuitive, as the amplitude of the acoustic pulse depends on the temperature increase during thermal excitation and the associated thermal expansion. As the pulse shape does not change, the frequency spectrum is the same with the exception of an offset.

The mechanical properties (density and Young’s modulus) affected the speed of sound in the material. It can be seen that the mechanical parameters influenced the converted sound amplitude and the frequency spectra in both the time and frequency domain.

The excitation parameters (laser, respectively, heat pulse duration and the optical absorption coefficient) are shown in Figure A6 and Figure A7. The pulse duration had a significant effect on the frequency bandwidth as well as the elongation. It should be noted that when varying the pulse duration, the introduced thermal power was kept constant in the simulation and therefore the pulse energy increases at longer pulse durations.

For the variation of the attenuation of the optical material, the inserted thermal power was kept constant for all absorption coefficients.

On the basis of the time signals and spectra shown in Figure A1, Figure A2, Figure A3, Figure A4, Figure A5, Figure A6 and Figure A7 it was possible to derive various dependencies between the sum of the squared elongation $s_{ac}^{2}$, the standard deviation σ and the amplitude *A* of the Gaussian fit in the linear scaled spectrum. The dependencies are visualised in the following figures and summarised in Formulas (19)–(21) in Section 3.1.
